# 10th Anniversary of Plants—Recent Advances and Further Perspectives

**DOI:** 10.3390/plants12081696

**Published:** 2023-04-18

**Authors:** Milan Stanković

**Affiliations:** Department of Biology and Ecology, Faculty of Science, University of Kragujevac, Radoja Domanovića 12, 34000 Kragujevac, Serbia; mstankovic@kg.ac.rs

Published for the first time in 2012, *Plants* will celebrate its 10th anniversary. To mark this significant milestone and celebrate the achievements made throughout the years, we intend to publish a Special Issue entitled “10th Anniversary of *Plants*—Recent Advances and Perspectives”. In the past decade, the continuous support of the authors, editors, and reviewers, as well as the readers, has resulted in noteworthy success and the achievement of a common goal, as well as the sustained reputation of *Plants* in the world of science. In parallel with the development of our journal, great success has also been achieved across the field of plant science itself, from the molecular to the ecosystem level, and many new findings are based on new methodological approaches. Apart from the fact that this Special Issue will serve as a celebration of the anniversary, it should also serve as a guide for discoveries in plant science and thus for the development of the journal.

This anniversary Special Issue contains 101 papers, the majority comprising articles (77 papers), followed by reviews (20 papers), communications (3 papers), and one protocol. The number of citations for this 10th anniversary Special Issue papers should be emphasized on this occasion; in the first year, this value has already reached almost 400, thereby confirming the importance and scientific impact of this collection as well as predicting an enviable future. Over 600 authors from all over the world contributed to the published papers of this Special Issue. Considering the satisfactory quality and diversity of the submissions, seven papers received the “featured” status, including four articles [1,2,3,4] and two reviews [5,6]. This status is awarded under very strict criteria defined by the publication policy of *Plants*. “Feature papers represent the most advanced research with significant potential for high impact in the field. A Feature Paper should be a substantial original Article that involves several techniques or approaches, provides an outlook for future research directions and describes possible research applications. Feature papers are submitted upon individual invitation or recommendation by the scientific editors and must receive positive feedback from the reviewers.” The number of papers with this status in relation to the total number of papers indicates the enviable scientific quality and impact of this Special Issue. The *Plants* editorial team practices the “Editor’s Choice” option. “Editor’s Choice articles are based on recommendations by the scientific editors of MDPI journals from around the world. Editors select a small number of articles recently published in the journal that they believe will be particularly interesting to readers, or important in the respective research area. The aim is to provide a snapshot of some of the most exciting work published in the various research areas of the journal.” This status was deservedly awarded to four papers, two articles [7,8], one review paper [9], as well as one protocol-type paper in this Special Issue [10]. An indispensable opportunity was to recognize the best paper from the 10th anniversary Special Issue. This complex task was assigned to the editors in charge of the issue. Scientific rigor, significance, citation, and originality were assessed in detail for all papers in the Special Issue. After that, the paper with the highest marks was announced [11].

During the establishment of the 10th anniversary Special Issue of *Plants*, no specific topic was defined, but all potential papers which were within the aims and scope of *Plants* were considered, and, based on that, the Special Issue was entitled “10th Anniversary of *Plants*—Recent Advances and Further Perspectives”. The published papers are characterized by great diversity concerning the topics in plant sciences, reflecting recent developments and the main trends, particularly in plant molecular biology and physiology, genetics, and phytochemistry. Though a complex task, based on the main topic, objects of research, as well as the contribution of the results, it was possible to categorize the papers into several groups. One group of papers is dedicated to molecular biology and the physiology of plants, applying modern molecular methodological approaches, cell biology, microbiology, etc., where molecular and physiological processes, interactions, stress resistance, etc. are elucidated. This group includes articles [1,2,7,12,13,14,15,16,17,18,19,20,21,22,23,24,25,26], communications [27,28], and review papers [5,6,11,29,30,31,32,33,34] with significant results applicable both in science and practice. The biology and ecology of plant secondary metabolites, their identification, and biological and therapeutic activity from different aspects of the phytochemistry of edible, aromatic, medicinal, or potentially medicinal plants are represented in a significant number of articles [3,8,35,36,37,38,39,40,41,42,43,44,45,46,47,48,49], as well as review papers [9,50,51]. The morphology and systematics of plants, as well as taxonomic methods, are also the subject of significant articles [52,53,54,55,56] and one review paper [57]. A similar number of papers are devoted to the scientific and practical aspects of ecology and the environment, comprising several articles [4,58,59,60,61] and one review [62]. The diversity of the topics of the Special Issue indicates the significant representation of agricultural plants in the trends of plant science. Physiology, molecular biology, and genetics, with a special aspect on the biotechnological approach, are covered in an impressive number of papers, such as articles [63,64,65,66,67,68,69,70,71,72,73,74,75,76,77,78,79,80,81,82,83,84,85,86,87,88,89,90,91,92,93,94], communications [95], and reviews [96,97,98,99,100,101], as well as the protocol described [10].

The abovementioned information illustrates the twofold significance of this Special Issue. It is a culmination of ten years of efforts to improve the quality of *Plants* and, at the same time, serves as a general overview of the achievements of plant sciences in the current period. I would like to take this opportunity to thank all the authors on behalf of the editorial office, especially for their interest in participating and willingness to share their experiences in science, as well as for the high-quality contributions submitted. We would like to thank the numerous reviewers for their valuable comments, which contributed to the quality of the published articles and thus to the overall quality of the Special Issue.

In terms of gratitude, it is imperative to note that the realization of the Special Issue was only possible thanks to the cooperation and dedication of the Special Issue editorial team—Prof. Dr. Milan Stanković (University of Kragujevac, Kragujevac, Serbia), Prof. Dr. Paula Baptista (Mountain Research Centre—CIMO, Bragança, Portugal), and Prof. Dr. Petronia Carillo (University of Campania Luigi Vanvitelli, Caserta, Italy). I would also like to thank the *Plants* editorial office, especially Ms. Sumi Sun, for their collaboration and guidance during the initiation, review, and editing process of the issue.

It has been my great pleasure to invest time and effort in editing this special issue, as well as to contribute over the past decade in the positions of assistant and associate editor of *Plants*.
